# Minimally invasive epicardial left ventricular lead placement in a case of massive pleural adhesion

**DOI:** 10.1186/1749-8090-9-70

**Published:** 2014-04-10

**Authors:** Hans-Stefan Hofmann, Michael Ried, Zsolt Sziklavari

**Affiliations:** 1 Department of Thoracic Surgery, Hospital Barmherzige Brüder Regensburg, Prüfeningerstraße 86, 93049 Regensburg, Germany; 2 Department of Thoracic Surgery, University Regensburg, Franz-Josef-Strauss-Allee 11, 93053 Regensburg, Germany

**Keywords:** Wound retractor, Left ventricular (LV) lead implantation, Video-assisted thoracoscopic surgery, Cardiac resynchronisation therapy

## Abstract

**Background:**

In cases of intravenous placement failure of the left ventricular (LV) lead for cardiac resynchronisation therapy (CRT) and obliteration of the left pleural space, the alternative approach of transthoracic placement by video-assisted thoracoscopic surgery (VATS) is difficult and not commonly practiced.

**Methods:**

Here, we present a simple technique for transthoracic introduction of an epicardial LV lead using a wound retractor (ALEXIS®) in a patient with heart failure. This wound retractor enables atraumatic tissue retraction without rib spreading, an optimal direct view in the pleural space for surgical pleurolysis and a high degree of safety for the patient.

**Results:**

No perioperative complications occurred. The tube drainage was removed on the second postoperative day, and the patient was discharged on the third postoperative day.

**Conclusions:**

The decided advantage of this new method is the lack of any need for rib spreading using a mechanical retractor. Especially in patients with a history of open-heart surgery (including internal mammary artery bypass grafting and/or revascularisation of the left lateral wall) or known pleural adhesions (e.g., pleuritis or lung operations), the described technique provides a rapid and save access with minimal surgical effort and greater safety.

## Background

Cardiac resynchronisation therapy (CRT) is a proven adjuvant therapeutic option for patients with severe and drug-refractory heart failure with significant intra- and interventricular dyssynchrony that results in considerable symptomatic improvement and increased survival rates for many patients. In the early phases of CRT, patients (e.g., the Ventak CHF trial) received a triple-chamber biventricular implantable cardioverter-defibrillator (ICD) with a transvenous right ventricular lead and a left ventricular (LV) lead placed via thoracotomy. Although substantial progress was made with the intravasal placement of the LV lead through a tributary vein of the coronary sinus (CS), this approach imposes technical constraints different from conventional epi- or endocardial ventricular pacing. For example, this technique involves the catheterisation of the CS and one of its tributaries, as well as stabilisation of the lead for long-term epicardial pacing. In addition, retrograde transvenous lead implantation may be impossible in some patients with aberrant anatomy. In these settings, successful lead placement has required innovative surgical approaches. Case series detailing the successful placement of the epicardial LV lead using minimally invasive procedures, such as video-assisted thoracoscopic surgery (VATS), have been presented [1,2]. However, in patients with previous thoracic operations and excessive pleural and pericardial adhesions, VATS techniques are complicated. In this report, we demonstrate a new method for minimally invasive epicardial LV lead placement without the need for classical (mini)thoracotomy despite the presence of pleural adhesions.

## Methods

The Ethics Commision at the Krankenhaus der Barmherzigen Brüder Regensburg approved this Study. In a 70-year-old patient with a progressive course of heart insufficiency and the indication for resynchronisation therapy (QRS-Duration 176 ms and the presence of left bundle branch block), the intravenous LV lead placement approach failed due to venous occlusion. It was a generator Promote® 3211-36Q (ST. Jude Medical) implanted.

The decision was made to proceed with LV lead placement using VATS with unilateral intubation. This operation was performed on the fourth postinterventional day. Due to a previous sternotomy (coronary artery bypass surgery 5 years prior) and the requirement for postoperative drainage management on the left side, the patient developed massive pleural adhesion, such that a classical VATS procedure was not possible.

**Figure 1:**
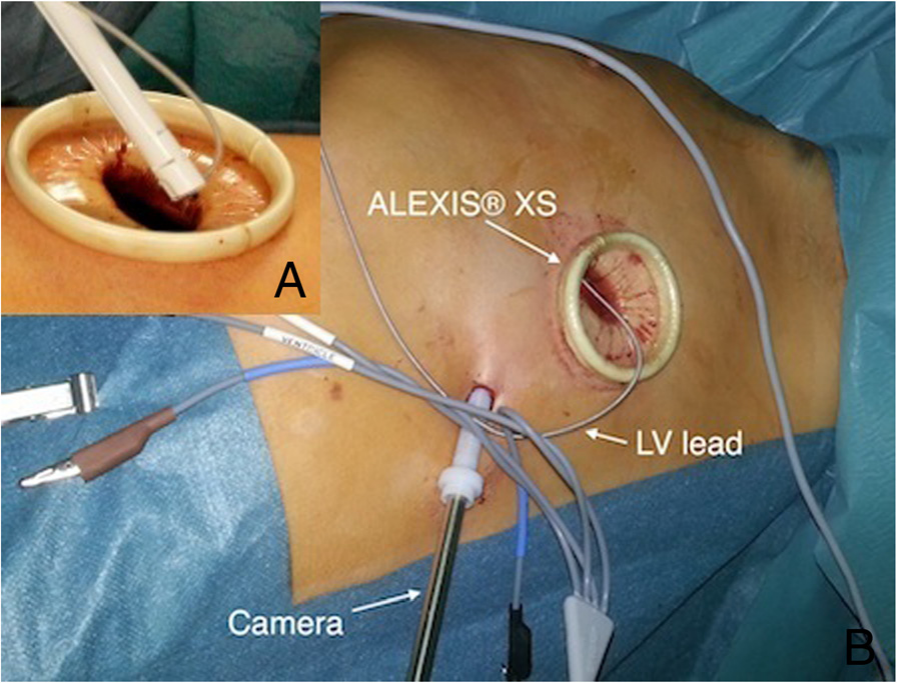
Intraoperative procedure; A: insertion of the LV lead with a lead introducer through the ALEXIS® retractor, B: overview of the operating field.

Therefore, we switched the procedure intraoperatively using the ALEXIS® XS wound protector/retractor (Applied Medical, Rancho Santa Margarita, CA). This disposable, single-use device consists of an elastic polymer membrane formed into the shape of a cylinder, with two semirigid polymer rings attached to each end of the cylinder. After a 3-cm-long skin incision (5^th^ intercostal space, midaxillary line) and division of the intercostal muscle were performed, the ALEXIS® retractor was positioned. The ALEXIS® wound protector/retractor provides 360 degrees of circumferential atraumatic retraction with a sufficiently wide opening of the pleural cavity. We then performed adhesiolysis via a semi-VATS procedure. Following pleurolysis of the parietal pleura, the camera was moved to an installed port in the 7^th^ intercostal space, posterior axillary line. Addition preparation was performed using video-assisted technology. Following pericardiotomy (1 cm in length), the posterolateral wall of the LV was exposed. The sutureless LV lead (Myodex, Bipolar 1084 T/54 St. Jude Medical, Inc.) was then screwed directly into the wall of the LV with a lead introducer through the ALEXIS® retractor (Figure 1). After confirming adequate pacing thresholds, a loop of the lead was left loose in the thoracic cavity, and the proximal end was passed through the chest wall to the ICD pocket for connection. We used the attached tunnelling instrument to pass the electrode via the subcutaneous tissues to the generator pocket. The lung was slowly ventilated under visualisation to ensure the lead position. A chest tube was placed through the mini incision to prevent postoperative effusions and pneumothorax.

## Results and discussion

No perioperative complications occurred. The tube drainage was removed on the second postoperative day, and the patient was discharged on the third postoperative day. The postoperative electrocardiogram showed an improvement in the QRS-Duration with 150 ms and the ventricular contractions were synchronous.

## Conclusions

Plastic wound retractors permit access to intra-abdominal/thoracic organs through an incision without the need for other mechanical retractors. These retractors may also act as a barrier to bacterial translocation from the abdominal/pleural cavity to the wound. In thoracic surgery, these retractors are used for video-assisted thoracoscopic pulmonary resections and for the treatment of pleural empyema [3,4]. Here, a new application for plastic wound retractors (the ALEXIS® wound protector/retractor) in managing epicardial LV lead implantation is described.

The use of VATS with two ports was reported to represent an excellent alternative procedure for epicardial lead implantation in classical open thoracotomy [1,2]. This alternative technique can be used in cases of failed standard transvenous LV lead placement. However, the presence of pleural and/or pericardial adhesions can hinder the VATS procedure and even force conversion to thoracotomy. In these cases, we recommend the use of the ALEXIS® retractor. The decided advantage of this new method is the lack of any need for rib spreading using a mechanical retractor. Especially in patients with a history of open-heart surgery (including internal mammary artery bypass grafting and/or revascularisation of the left lateral wall) or known pleural adhesions (e.g., pleuritis or lung operations), the described technique provides rapid treatment with minimal surgical effort and greater safety.

## Abbreviations

LV: Left ventricular; CRT: Cardiac resynchronisation therapy; VATS: Video-assisted thoracoscopic surgery; ICD: Implantable cardioverter-defibrillator.

## Competing interests

The authors declare that they have no competing interests.

## Authors’ contributions

HH, and MR participated in the design of the study. ZS and HH participated in the sequence alignment and drafted the manuscript. MR conceived of the study and participated in its design and coordination. HH participated in surgery. All authors read and approved the final manuscript.
